# Scoping reviews in medical education: A scoping review

**DOI:** 10.1111/medu.14431

**Published:** 2020-12-30

**Authors:** Lauren A. Maggio, Kelsey Larsen, Aliki Thomas, Joseph A. Costello, Anthony R. Artino

**Affiliations:** ^1^ Department of Medicine Uniformed Services University of the Health Sciences Bethesda MD USA; ^2^ Department of Politics, Security, and International Affairs University of Central Florida Orlando FL USA; ^3^ School of Physical and Occupational Therapy Institute of Health Sciences Education Faculty of Medicine McGill University Montreal QC Canada; ^4^ Uniformed Services University of the Health Sciences Bethesda MD USA; ^5^ Department of Health, Human Function, and Rehabilitation Sciences The George Washington University School of Medicine and Health Sciences Washington DC USA

## Abstract

**Objectives:**

Over the last two decades, the number of scoping reviews in core medical education journals has increased by 4200%. Despite this growth, research on scoping reviews provides limited information about their nature, including how they are conducted or why medical educators undertake this knowledge synthesis type. This gap makes it difficult to know where the field stands and may hamper attempts to improve the conduct, reporting and utility of scoping reviews. Thus, this review characterises the nature of medical education scoping reviews to identify areas for improvement and highlight future research opportunities.

**Method:**

The authors searched PubMed for scoping reviews published between 1/1999 and 4/2020 in 14 medical education journals. The authors extracted and summarised key bibliometric data, the rationales given for conducting a scoping review, the research questions and key reporting elements as described in the PRISMA‐ScR. Rationales and research questions were mapped to Arksey and O'Malley's reasons for conducting a scoping review.

**Results:**

One hundred and one scoping reviews were included. On average, 10.1 scoping reviews (SD = 13.1, median = 4) were published annually with the most reviews published in 2019 (n = 42). Authors described multiple reasons for undertaking scoping reviews; the most prevalent being to summarise and disseminate research findings (n = 77). In 11 reviews, the rationales for the scoping review and the research questions aligned. No review addressed all elements of the PRISMA‐ScR, with few authors publishing a protocol (n = 2) or including stakeholders (n = 20). Authors identified shortcomings of scoping reviews, including lack of critical appraisal.

**Conclusions:**

Scoping reviews are increasingly conducted in medical education and published by most core journals. Scoping reviews aim to map the depth and breadth of emerging topics; as such, they have the potential to play a critical role in the practice, policy and research of medical education. However, these results suggest improvements are needed for this role to be fully realised.

## Abstract 2

Over the last 20 years, the number of #MedEd reviews has grown >2600% (4200% for scoping reviews). To explore this growth, Maggio et al. conducted a scoping review of 101 scoping reviews across 14 journals.

## 1 INTRODUCTION

Over the last two decades, the number of knowledge syntheses published in core medical education journals has increased by 2620%.^1^ Among these knowledge syntheses, there has been an even steeper rise in the number of scoping reviews published, with that number increasing by 4200%. The growth of scoping reviews is prompting discussions among scholars regarding the role of scoping reviews in the field and their potential to influence educational practices, policies and future research. However, despite this growth, the extant research on scoping reviews provides limited information about their nature, including how they are conducted, if they are funded, or why medical educators decide to undertake this type of knowledge synthesis in the first place. This lack of direct insight makes it difficult to know where the field stands and may hamper attempts to take evidence‐informed steps to improve the conduct, reporting and utility of scoping reviews in medical education.

Scoping reviews are often cast as publications that ‘map’ the depth and breadth of the literature in a field.^2^, ^3^ Through such synthetic mapping, authors describe the main concepts that underpin a topic and can illuminate gaps in the literature. Scoping reviews are generally driven by broad, exploratory research questions and typically incorporate studies that employ a variety of research designs.^4^, ^5^ In their seminal article outlining a model for scoping reviews, Arksey and O'Malley^2^ described a six‐step framework for conducting scoping reviews. These steps include the following: (a) identifying the research question, (b) identifying relevant studies, (c) selecting the studies to be included, (d) charting the data, (e) collating, summarising and reporting results and (f) consultation with stakeholders. Over time, scholars have suggested modifications to the steps.^4^, ^5^, ^6^, ^7^ Some of these modifications are captured in the Preferred Reporting Items for Systematic Reviews and Meta‐analysis Extension for Scoping Reviews (PRISMA‐ScR),^8^ the first reporting guideline specific to scoping reviews.

Similar to medical education, the number of scoping reviews published in the health sciences is also on the rise.^9^, ^10^ To characterise these scoping reviews, researchers have recently authored discipline‐specific^11^ and cross‐disciplinary^3^, ^12^ scoping reviews of scoping reviews. Collectively, these scoping reviews have identified methodological shortcomings and a need for improved scoping review reporting. While these studies are valuable, two are now several years old and the most recent review focuses solely on rehabilitation medicine, thus providing limited information on current approaches specific to medical education. What is more, the multi‐disciplinary nature of medical education research suggests there may be variability in how researchers approach scoping reviews in our field, the topics they choose to review, and their purposes for using a scoping review. These differences warranted further exploration, which we undertook in the present scoping review.

As a relatively new field that includes researchers from a variety of backgrounds and research traditions, we believe that medical education scoping reviews are not immune to the methodological and reporting concerns found in other disciplines.^13^ Thus, we propose that there is value in specifically examining scoping reviews in medical education and assert that in light of their exponential growth rate, the time is now to undertake such an analysis. In doing so, we hope to identify areas for improvement in the conduct and reporting of scoping reviews in medical education, thereby helping to ensure that those produced are relevant to and practical for application in the field (eg, useful for mapping the literature of a topic or identifying gaps in the literature). We do not aim to revisit the usefulness of scoping reviews or their methodological and epistemological considerations, as others have done in previous papers.^2^, ^13^ Rather, in the study reported here, we aim to characterise the extent, range and nature of scoping reviews published in medical education journals in order to identify areas for improving their conduct and reporting, and to highlight future research opportunities.

## 2 METHODS

Guided by the framework presented by Arksey and O'Malley^2^ as updated by Levac et al,^5^ we conducted a scoping review of medical education scoping reviews to examine and characterise the extent, range and nature of scoping reviews in core medical education journals.

### 2.1 Identifying the research question

This scoping review is a component of a larger bibliometric analysis conducted by members of the author team. In the larger analysis, we broadly characterised knowledge syntheses in a core set of medical education journals and observed exponential growth in the number of scoping reviews.^1^ This observation prompted three follow‐on questions: (a) What are the characteristics of the scoping reviews and how can they be improved?; (b) What rationales do authors provide for undertaking a scoping review?; and (c) How do authors report the details of their scoping reviews?

### 2.2 Identifying relevant studies

In the present study, we identified scoping reviews published during the time frame of the original study (1999‐2019)^1^ plus those reviews published in the first four months of 2020. On 26 March 2020 JC, an information scientist, queried PubMed using a combination of keywords and controlled vocabulary terms (See Appendix S1 for complete searches).^1^ He reran this search on 27 April 2020 to capture any new citations. All retrieved citations and their metadata (eg, abstract, author names) were managed in GoogleSheets.^14^ On 21 May, 2020, we obtained from Web of Science the number of times each review had been cited.

Searches were limited to 14 journals previously identified as core medical education titles,^15^, ^16^ including: *Academic Medicine, Advances in Health Sciences Education, BMC Medical Education, Canadian Medical Education Journal, Clinical Teacher, International Journal of Medical Education, Advances in Medical Education and Practice, Journal of Graduate Medical Education, Medical Education, Medical Education Online, Medical Teacher, Perspectives on Medical Education, Teaching and Learning in Medicine*, and *The Journal of Continuing Education in the Health Professions*. In addition to having been previously identified by researchers as core medical education journals,^15^, ^16^ we selected these titles because they are indexed in the Web of Science. This indexing enabled us to obtain citation data for individual reviews. Additionally, we erred on the side of search strategy accuracy by targeting this curated set of education‐focused publications. Thus, we did not search PubMed broadly using keywords, which would have opened our search to all biomedical journals. As such, our search strategy ensured that retrieved studies were focused on medical education, especially because the indexing of medical education content is not comprehensive. Lastly, due to the nature of our research questions, our search was restricted only to scoping reviews.

We assembled a research team with expertise in knowledge synthesis methodology, information science, and medical education to guide the overall conduct of the review and our interpretation of the results. All team members had conducted previous scoping reviews.

### 2.3 Selecting the studies to be included

To select studies for inclusion, we used an iterative approach. LM and JC independently reviewed the titles and abstracts of all citations. To facilitate calibration, they met four times during the process with the first meeting focused on creating a shared understanding of the criteria and then in the three subsequent meetings comparing selected citations and discussing any discrepancies. AA was available to facilitate any coding disagreements. Articles were included if they described the conduct of a scoping review. This initial determination was generally made based on the presence of the word ‘scoping’ or variants thereof and the mention of Arksey and O'Malley^2^ and Levac^5^; however, articles in which authors discussed scoping reviews as a methodological approach, but that did not describe undertaking an actual scoping review, were excluded.

### 2.4 Charting the data

We created a data extraction tool that included and expanded upon the 22 items from the PRISMA‐ScR checklist.^8^ The extraction tool also included, but was not limited to, questions about the review's population, authors' rationale for undertaking the review, and the stated research questions or aims. (See https://doi.org/10.6084/m9.figshare.12699698.v2 for the data charting tool completed for each included study).

LM and JC piloted the data extraction tool by independently reviewing seven reviews and then comparing results. The extraction tool was modified based on the pilot and then used to extract data from the remaining full texts of articles. LM and JC independently extracted data from all included articles and met three times to discuss any discrepancies. AA was again available to discuss any coding disagreements and serve as tiebreaker.

### 2.5 Collating, summarising and reporting results

We calculated descriptive statistics using GoogleSheets^14^ to describe review characteristics. To describe the authors' rationales for undertaking a scoping review and their research questions, we conducted a thematic analysis.^17^ To begin, LM and JC familiarised themselves with the data through multiple readings. During an initial round of open coding, they independently identified the relevance of the four reasons proposed by Arksey and O'Malley for conducting a scoping review and discussed these reasons via conference call. These reasons include: (a) to examine the extent, range and nature of research activity in a given area; (b) to determine the value of undertaking a full systematic review; (c) to summarise and disseminate research findings; and (d) to identify gaps in the existing body of literature.^2^ Based on a subsequent whole‐team call, we decided to use these four reasons as a priori codes while remaining open to additional rationales for preliminary coding. LM and JC independently coded all rationales and research questions and then met with a third author, KL. The meeting focused on cross‐checking agreement on the overall coding of rationales and research questions and resolving any disagreements through discussion.

### 2.6 Undertaking consultation

We shared our preliminary findings with seven stakeholders to understand if and in what ways our findings resonated with their experiences conducting scoping reviews. Stakeholders were authors of scoping reviews (n = 6), editors of medical education journals (n = 2) and faculty members in health professions education graduate programmes (n = 2). Stakeholders were asked to review our results and suggest topics for discussion and future research. All seven stakeholders agreed that our findings corresponded with their experiences; five provided suggestions for interpretation, which we incorporated into our discussion.

## 3 RESULTS

We included 101 studies (See Appendix S2 for a diagram of the inclusion process).^18^, ^19^, ^20^, ^21^, ^22^, ^23^, ^24^, ^25^, ^26^, ^27^, ^28^, ^29^, ^30^, ^31^, ^32^, ^33^, ^34^, ^35^, ^36^, ^37^, ^38^, ^39^, ^40^, ^41^, ^42^, ^43^, ^44^, ^45^, ^46^, ^47^, ^48^, ^49^, ^50^, ^51^, ^52^, ^53^, ^54^, ^55^, ^56^, ^57^, ^58^, ^59^, ^60^, ^61^, ^62^, ^63^, ^64^, ^65^, ^66^, ^67^, ^68^, ^69^, ^70^, ^71^, ^72^, ^73^, ^74^, ^75^, ^76^, ^77^, ^78^, ^79^, ^80^, ^81^, ^82^, ^83^, ^84^, ^85^, ^86^, ^87^, ^88^, ^89^, ^90^, ^91^, ^92^, ^93^, ^94^, ^95^, ^96^, ^97^, ^98^, ^99^, ^100^, ^101^, ^102^, ^103^, ^104^, ^105^, ^106^, ^107^, ^108^, ^109^, ^110^, ^111^, ^112^, ^113^, ^114^, ^115^, ^116^, ^117^, ^118^ On average 10.1 scoping reviews (SD = 13.1, median = 4, range 0‐42) were published annually (See Appendix S3) with the most published in 2019 (n = 42; 41.6%). The first scoping review in our sample was published in 2011. Scoping reviews were featured in 13 of the 14 journals with *Academic Medicine* (n = 28, 27.7%),^18^, ^24^, ^49^, ^52^, ^55^, ^58^, ^61^, ^62^, ^68^, ^72^, ^73^, ^76^, ^90^, ^92^, ^94^, ^97^, ^100^, ^104^, ^112^, ^115^, ^116^ *Medical Education* (n = 18, 17.8%)^20^, ^65^, ^66^, ^67^, ^77^, ^103^, ^108^, ^109^, ^110^, ^111^ and *BMC Medical Education* (n = 16, 15.8%)^19^, ^26^, ^29^, ^39^, ^45^, ^50^, ^51^, ^58^, ^69^, ^70^, ^84^, ^85^, ^87^, ^96^, ^105^, ^113^ publishing the most. *Clinical Teacher* did not publish any scoping reviews during this time period. Thirty‐eight scoping reviews (37.6%)^19^, ^26^, ^28^, ^51^, ^53^, ^58^, ^60^, ^62^, ^65^, ^66^, ^70^, ^72^, ^73^, ^74^, ^75^, ^87^, ^88^, ^90^, ^92^, ^96^, ^97^, ^100^, ^101^, ^102^, ^105^, ^106^, ^109^, ^110^, ^112^, ^117^ reported funding that supported the work with nearly half (39.5%)^41^, ^44^, ^49^, ^70^, ^72^, ^73^, ^75^, ^87^, ^88^, ^90^, ^92^, ^100^, ^101^, ^105^, ^115^ of those funded receiving public funds. All reviews synthesised journal articles, with 28.7% (n = 29) also including book chapters, grey literature, dissertations, websites, posters and conference proceedings.^19^, ^23^, ^51^, ^56^, ^57^, ^59^, ^61^, ^63^, ^64^, ^71^, ^75^, ^76^, ^88^, ^92^, ^93^, ^95^, ^99^, ^104^, ^106^, ^108^, ^114^, ^117^ Of those that focused only on journal articles, multiple reviews limited inclusion to original research studies, thereby excluding commentaries, letters, editorials and review articles.

Scoping reviews were authored, on average, by 5.3 authors per review (SD = 2.9, median = 5, range 1‐17). A single review featured one author only.^111^ Lead authors were based in 16 countries with the majority in Canada (n = 31, 30.7%),^18^, ^19^, ^22^, ^62^, ^71^, ^80^, ^81^, ^86^, ^94^, ^95^, ^97^, ^98^, ^99^, ^100^, ^102^, ^103^, ^106^, ^108^, ^112^, ^113^, ^114^, ^115^, ^116^, ^117^, ^118^ the United States (n = 27, 26.7%)^32^, ^35^, ^48^, ^49^, ^52^, ^53^, ^54^, ^55^, ^59^, ^61^, ^68^, ^72^, ^73^, ^74^, ^77^, ^82^, ^91^, ^92^, ^104^, ^109^ and Australia (n = 9, 8.9%).^30^, ^46^, ^58^, ^67^, ^75^, ^79^, ^89^, ^93^, ^96^ In 23 reviews (22.8%), authors described their team members' backgrounds and expertise in relation to their scoping review.^20^, ^21^, ^22^, ^60^, ^66^, ^67^, ^71^, ^72^, ^73^, ^74^, ^76^, ^86^, ^88^, ^97^, ^99^, ^104^, ^112^ For example, authors of a scoping review on parenthood noted that they ‘had knowledge and experience of parenthood during GME (JAB, SWS), literature reviews (ALB, KEE, SM) and information management (ALB)’.^44^ Doctoral students in health professions education led ten studies (9.9%).^23^, ^33^, ^46^, ^48^, ^60^, ^71^, ^74^, ^76^, ^88^, ^99^

While not all scoping reviews had available citation data, those that did (n = 89) were cited, on average, 6.4 times (SD = 11.7, median = 2, range 0‐61).^19^, ^20^, ^21^, ^22^, ^23^, ^25^, ^26^, ^27^, ^28^, ^29^, ^30^, ^31^, ^32^, ^34^, ^35^, ^36^, ^37^, ^39^, ^40^, ^44^, ^45^, ^46^, ^48^, ^49^, ^50^, ^51^, ^52^, ^53^, ^54^, ^55^, ^56^, ^57^, ^58^, ^60^, ^61^, ^62^, ^63^, ^64^, ^65^, ^66^, ^67^, ^68^, ^69^, ^70^, ^71^, ^73^, ^74^, ^75^, ^76^, ^77^, ^78^, ^79^, ^80^, ^81^, ^83^, ^84^, ^85^, ^86^, ^87^, ^88^, ^89^, ^90^, ^92^, ^93^, ^94^, ^95^, ^96^, ^97^, ^98^, ^99^, ^100^, ^101^, ^102^, ^103^, ^104^, ^105^, ^106^, ^107^, ^108^, ^109^, ^110^, ^111^, ^112^, ^113^, ^114^, ^115^, ^116^, ^117^, ^118^ Eighteen articles (17.8%) had not been cited^19^, ^20^, ^21^, ^22^, ^23^, ^37^, ^39^, ^45^, ^53^, ^54^, ^78^, ^88^; of those, 10 (55.6%)^19^, ^20^, ^21^, ^22^, ^23^, ^54^ were published after 2019. (See the Appendix S4 for the 10 most cited scoping reviews).

### 3.1 Rationale for scoping reviews

Eighty‐eight (87.1%)^18^, ^19^, ^20^, ^21^, ^22^, ^23^, ^24^, ^25^, ^26^, ^27^, ^28^, ^30^, ^31^, ^32^, ^33^, ^35^, ^36^, ^37^, ^39^, ^40^, ^41^, ^43^, ^44^, ^45^, ^48^, ^49^, ^50^, ^51^, ^52^, ^53^, ^54^, ^55^, ^56^, ^58^, ^59^, ^60^, ^61^, ^62^, ^63^, ^64^, ^65^, ^66^, ^67^, ^68^, ^70^, ^72^, ^73^, ^74^, ^75^, ^76^, ^77^, ^78^, ^79^, ^80^, ^81^, ^82^, ^85^, ^86^, ^87^, ^88^, ^89^, ^90^, ^91^, ^92^, ^93^, ^94^, ^95^, ^96^, ^98^, ^99^, ^100^, ^101^, ^103^, ^104^, ^105^, ^106^, ^107^, ^108^, ^109^, ^110^, ^111^, ^112^, ^113^, ^114^, ^115^, ^116^, ^118^ authors described rationales for selecting a scoping review methodology, with most referencing multiple rationales (n = 82; 93.1%).^18^, ^19^, ^20^, ^21^, ^22^, ^23^, ^24^, ^25^, ^26^, ^27^, ^28^, ^30^, ^31^, ^32^, ^33^, ^35^, ^36^, ^37^, ^39^, ^40^, ^43^, ^44^, ^45^, ^48^, ^49^, ^50^, ^51^, ^52^, ^53^, ^54^, ^56^, ^58^, ^59^, ^60^, ^61^, ^62^, ^63^, ^65^, ^66^, ^67^, ^68^, ^70^, ^72^, ^74^, ^75^, ^76^, ^77^, ^78^, ^79^, ^80^, ^81^, ^82^, ^85^, ^86^, ^87^, ^88^, ^89^, ^90^, ^91^, ^92^, ^93^, ^95^, ^96^, ^98^, ^99^, ^100^, ^101^, ^103^, ^104^, ^105^, ^106^, ^107^, ^108^, ^109^, ^110^, ^111^, ^112^, ^113^, ^114^, ^115^, ^116^, ^118^ There were, on average, 2.6 rationales stated per review (SD = 1.4, median = 3;). The most often stated rationales were: to summarise and disseminate research findings (n = 77; 87.5%)^18^, ^19^, ^20^, ^21^, ^22^, ^23^, ^24^, ^25^, ^26^, ^27^, ^28^, ^30^, ^31^, ^32^, ^33^, ^36^, ^37^, ^39^, ^40^, ^43^, ^44^, ^45^, ^48^, ^49^, ^50^, ^51^, ^52^, ^53^, ^54^, ^56^, ^58^, ^59^, ^60^, ^63^, ^65^, ^66^, ^68^, ^72^, ^74^, ^75^, ^76^, ^77^, ^78^, ^79^, ^81^, ^82^, ^85^, ^86^, ^87^, ^88^, ^89^, ^90^, ^91^, ^92^, ^93^, ^95^, ^96^, ^99^, ^100^, ^101^, ^103^, ^104^, ^105^, ^106^, ^107^, ^108^, ^109^, ^110^, ^111^, ^112^, ^113^, ^114^, ^115^, ^116^; to examine the extent, range, and nature of research activity in a given area (n = 74; 84.1%)^18^, ^19^, ^20^, ^21^, ^22^, ^24^, ^25^, ^26^, ^27^, ^30^, ^31^, ^32^, ^33^, ^35^, ^36^, ^37^, ^39^, ^40^, ^43^, ^44^, ^48^, ^49^, ^50^, ^51^, ^52^, ^53^, ^54^, ^56^, ^59^, ^60^, ^61^, ^62^, ^63^, ^65^, ^66^, ^68^, ^72^, ^74^, ^75^, ^76^, ^77^, ^78^, ^79^, ^81^, ^82^, ^84^, ^85^, ^86^, ^87^, ^88^, ^89^, ^90^, ^91^, ^92^, ^93^, ^95^, ^96^, ^99^, ^100^, ^101^, ^103^, ^104^, ^105^, ^106^, ^108^, ^109^, ^110^, ^111^, ^112^, ^113^, ^114^, ^115^, ^116^, ^118^; and to contend with the nature of the study topic or available literature (n = 46; 52.3%).^19^, ^21^, ^23^, ^24^, ^25^, ^26^, ^39^, ^40^, ^41^, ^45^, ^50^, ^54^, ^55^, ^58^, ^59^, ^60^, ^61^, ^62^, ^63^, ^64^, ^68^, ^73^, ^75^, ^77^, ^80^, ^82^, ^86^, ^92^, ^94^, ^99^, ^101^, ^104^, ^105^, ^106^, ^107^, ^109^, ^112^, ^113^, ^114^, ^116^ See Appendix S5 for all rationales.

### 3.2 Research questions/Study aims

Ninety‐eight authors (97.0%) included research questions and/or aims.^18^, ^19^, ^20^, ^21^, ^22^, ^23^, ^24^, ^25^, ^26^, ^27^, ^28^, ^29^, ^30^, ^31^, ^32^, ^33^, ^34^, ^35^, ^36^, ^37^, ^38^, ^39^, ^41^, ^42^, ^43^, ^44^, ^45^, ^46^, ^47^, ^48^, ^49^, ^50^, ^51^, ^52^, ^53^, ^54^, ^55^, ^56^, ^57^, ^58^, ^59^, ^60^, ^61^, ^62^, ^63^, ^64^, ^65^, ^66^, ^67^, ^68^, ^69^, ^70^, ^71^, ^72^, ^73^, ^74^, ^75^, ^76^, ^77^, ^78^, ^79^, ^80^, ^81^, ^82^, ^84^, ^85^, ^86^, ^87^, ^88^, ^89^, ^90^, ^92^, ^93^, ^94^, ^95^, ^96^, ^97^, ^98^, ^99^, ^100^, ^101^, ^102^, ^103^, ^104^, ^105^, ^106^, ^107^, ^108^, ^109^, ^110^, ^111^, ^112^, ^113^, ^114^, ^115^, ^116^, ^117^, ^118^ Authors put forth, on average, 2.4 research questions or aims per review (SD = 1.0, median = 2, range 0‐5). Similar to their rationales for conducting a scoping review, authors' research questions or aims were attempting to: summarise and disseminate research findings (n = 89; 90.8%)^18^, ^19^, ^20^, ^21^, ^22^, ^23^, ^24^, ^25^, ^26^, ^27^, ^28^, ^29^, ^30^, ^31^, ^32^, ^34^, ^35^, ^36^, ^37^, ^38^, ^39^, ^41^, ^42^, ^43^, ^44^, ^45^, ^46^, ^47^, ^48^, ^49^, ^50^, ^51^, ^52^, ^53^, ^54^, ^55^, ^56^, ^57^, ^58^, ^59^, ^60^, ^61^, ^62^, ^63^, ^64^, ^65^, ^66^, ^67^, ^68^, ^69^, ^71^, ^72^, ^73^, ^74^, ^76^, ^77^, ^78^, ^79^, ^80^, ^81^, ^82^, ^84^, ^85^, ^86^, ^87^, ^88^, ^92^, ^93^, ^94^, ^96^, ^97^, ^98^, ^99^, ^100^, ^101^, ^102^, ^103^, ^105^, ^106^, ^107^, ^109^, ^110^, ^111^, ^112^, ^113^, ^114^, ^116^, ^117^, ^118^; examine the extent, range and nature of research activity in a given area (n = 86; 87.8%)^18^, ^19^, ^20^, ^21^, ^22^, ^23^, ^24^, ^25^, ^26^, ^27^, ^28^, ^29^, ^30^, ^32^, ^34^, ^35^, ^36^, ^37^, ^38^, ^39^, ^41^, ^42^, ^43^, ^44^, ^45^, ^46^, ^47^, ^48^, ^49^, ^50^, ^51^, ^52^, ^53^, ^54^, ^55^, ^56^, ^57^, ^59^, ^60^, ^61^, ^62^, ^63^, ^64^, ^65^, ^66^, ^67^, ^68^, ^69^, ^71^, ^72^, ^73^, ^74^, ^76^, ^77^, ^78^, ^79^, ^80^, ^81^, ^82^, ^84^, ^85^, ^86^, ^87^, ^88^, ^92^, ^94^, ^96^, ^97^, ^99^, ^101^, ^102^, ^103^, ^104^, ^105^, ^106^, ^107^, ^109^, ^110^, ^111^, ^112^, ^113^, ^114^, ^115^, ^116^, ^117^, ^118^; and contend with the nature of the study topic or available literature (n = 18; 18.4%).^19^, ^24^, ^27^, ^30^, ^33^, ^37^, ^45^, ^48^, ^68^, ^75^, ^78^, ^82^, ^92^, ^108^, ^110^, ^112^, ^114^, ^118^

Although authors usually provided more rationales for selecting the scoping review methodology than they offered research questions, there was some alignment in our coding of the authors' rationales and their research questions/aims. In 11 reviews (10.9%), the rationales for conducting a scoping review and the research questions were in complete alignment, such that we coded each in the exact same way.^19^, ^24^, ^43^, ^49^, ^52^, ^65^, ^70^, ^76^, ^81^, ^85^, ^92^ In 65 studies (64.4%) there was overlap such that the research questions indicated a desire to summarise the literature and examine its nature, but the rationale for selecting a scoping review included additional rationales, such as going further to describe the need to deal with heterogeneity of the available literature.^18^, ^19^, ^20^, ^21^, ^22^, ^23^, ^24^, ^25^, ^26^, ^27^, ^28^, ^30^, ^31^, ^32^, ^35^, ^36^, ^37^, ^39^, ^43^, ^44^, ^45^, ^48^, ^49^, ^50^, ^51^, ^52^, ^53^, ^54^, ^56^, ^59^, ^60^, ^61^, ^62^, ^63^, ^65^, ^66^, ^68^, ^72^, ^74^, ^76^, ^77^, ^78^, ^79^, ^81^, ^82^, ^85^, ^86^, ^87^, ^88^, ^92^, ^96^, ^99^, ^101^, ^103^, ^105^, ^106^, ^107^, ^109^, ^110^, ^111^, ^112^, ^113^, ^114^, ^116^ For example, in one study, the authors described undertaking a scoping review to identify gaps in the research and clarify key concepts, as well as to clarify definitions of the concept; this aligned with, but went beyond, their research question, which was to describe the scope of the literature on the topic.^61^

### 3.3 Reporting in alignment with PRISMA‐ScR

Studies reported items from the PRISMA‐ScR to varying degrees, and none included all of the items. Thirteen reviews (12.9%)^29^, ^30^, ^57^, ^58^, ^63^, ^67^, ^68^, ^72^, ^73^, ^76^, ^88^, ^99^, ^102^ cited following the PRISMA,^119^ and five (5.0%) the PRISMA‐ScR.^8^, ^35^, ^36^, ^41^, ^52^, ^84^ Table 1 summarises the components of the PRISMA‐ScR present in the included scoping reviews. For details by study, see https://doi.org/10.6084/m9.figshare.12699698.v2.

**TABLE 1:** A summary of the presence of the PRISMA‐ScR checklist items in 101 scoping reviews in 14 core medical education journals

Checklist item	Number scoping reviews (%)	References
Study identified as a scoping review in the title	89 (88.1)	18, 19, 20, 21, 22, 23, 24, 25, 26, 27, 28, 29, 30, 31, 32, 33, 34, 35, 36, 37, 38, 39, 40, 41, 42, 43, 44, 45, 46, 47, 48, 49, 50, 51, 52, 53, 54, 55, 56, 58, 59, 60, 61, 62, 63, 64, 65, 66, 68, 69, 70, 71, 72, 73, 75, 77, 78, 79, 80, 82, 84, 85, 86, 87, 88, 90, 92, 93, 94, 96, 97, 99, 100, 101, 102, 103, 104, 105, 106, 107, 108, 110, 112, 113, 114, 115, 116, 117, 118
Includes a structured abstract	100 (99.0)	18, 19, 20, 21, 22, 23, 24, 25, 26, 27, 28, 29, 30, 31, 32, 33, 34, 35, 36, 37, 38, 39, 40, 41, 42, 43, 44, 45, 46, 47, 48, 49, 50, 51, 52, 53, 54, 55, 56, 58, 118
Describes the rationale for the review in the context of what is already known	88 (87.0)	18, 19, 20, 21, 22, 23, 24, 25, 26, 27, 28, 30, 31, 32, 33, 35, 36, 37, 39, 40, 41, 43, 44, 45, 48, 49, 50, 51, 52, 53, 54, 55, 56, 58, 59, 60, 61, 62, 63, 64, 65, 66, 67, 68, 70, 72, 73, 74, 75, 76, 77, 78, 79, 80, 81, 82, 84, 85, 86, 87, 88, 89, 90, 91, 92, 93, 94, 95, 96, 98, 99, 100, 101, 103, 104, 105, 106, 107, 108, 109, 110, 111, 112, 113, 114, 115, 116, 118
Explains why review questions/aims lend themselves to a scoping review approach	98 (97.0)	18, 19, 20, 21, 22, 23, 24, 25, 26, 27, 28, 29, 30, 31, 32, 33, 34, 35, 36, 37, 38, 39, 41, 42, 43, 44, 45, 46, 47, 48, 49, 50, 51, 52, 53, 54, 55, 56, 57, 58, 59, 60, 61, 62, 63, 64, 65, 66, 67, 68, 69, 70, 71, 72, 73, 74, 75, 76, 77, 78, 79, 80, 81, 82, 84, 85, 86, 87, 88, 89, 90, 92, 93, 94, 95, 96, 97, 98, 99, 100, 101, 102, 103, 104, 105, 106, 107, 108, 109, 110, 111, 112, 113, 114, 115, 116, 117, 118
Provides the questions and objectives being addressed	101 (100)	18, 19, 20, 21, 22, 23, 24, 25, 26, 27, 28, 29, 30, 31, 32, 33, 34, 35, 36, 37, 38, 39, 40, 41, 42, 43, 44, 45, 46, 47, 48, 49, 50, 51, 52, 53, 54, 55, 56, 57, 58, 59, 60, 61, 62, 63, 64, 65, 66, 67, 68, 69, 70, 71, 72, 73, 74, 75, 76, 77, 78, 79, 80, 81, 82, 83, 84, 85, 86, 87, 88, 89, 90, 91, 92, 93, 94, 95, 96, 97, 98, 99, 100, 101, 102, 103, 104, 105, 106, 107, 108, 109, 110, 111, 112, 113, 114, 115, 116, 117, 118
Indicates whether a review protocol exists	2 (2.0)	35, 36
Specifies characteristics of the sources of evidence used as eligibility criteria and provides rationale	98 (97.0)	18, 19, 20, 21, 22, 23, 24, 26, 27, 28, 29, 30, 31, 32, 33, 34, 35, 36, 37, 38, 39, 40, 41, 42, 43, 44, 45, 46, 47, 48, 49, 50, 51, 52, 53, 54, 55, 56, 57, 58, 59, 60, 61, 62, 63, 64, 65, 66, 67, 68, 69, 70, 71, 72, 73, 74, 75, 76, 77, 78, 79, 80, 81, 82, 84, 85, 86, 87, 88, 89, 90, 91, 92, 93, 94, 95, 96, 97, 98, 99, 100, 101, 102, 103, 104, 105, 106, 107, 109, 110, 111, 112, 113, 114, 115, 116, 117, 118
Presents all information sources searched	101 (100)	18, 19, 20, 21, 22, 23, 24, 25, 26, 27, 28, 29, 30, 31, 32, 33, 34, 35, 36, 37, 38, 39, 40, 41, 42, 43, 44, 45, 46, 47, 48, 49, 50, 51, 52, 53, 54, 55, 56, 57, 58, 59, 60, 61, 62, 63, 64, 65, 66, 67, 68, 69, 70, 71, 72, 73, 74, 75, 76, 77, 78, 79, 80, 81, 82, 83, 84, 85, 86, 87, 88, 89, 90, 91, 92, 93, 94, 95, 96, 97, 98, 99, 100, 101, 102, 103, 104, 105, 106, 107, 108, 109, 110, 111, 112, 113, 114, 115, 116, 117, 118
Includes the most recent search date	51 (50.5)	20, 22, 24, 27, 28, 29, 30, 31, 35, 36, 37, 41, 44, 45, 46, 50, 51, 52, 54, 55, 56, 57, 59, 60, 61, 62, 65, 66, 68, 71, 72, 73, 74, 75, 76, 78, 86, 87, 90, 91, 92, 94, 97, 100, 101, 102, 104, 110, 113, 115
Includes the full search strategy for at least one database such that it could be repeated	62 (61.4)	18, 19, 20, 21, 22, 23, 24, 35, 36, 37, 38, 39, 40, 44, 45, 46, 47, 50, 51, 52, 53, 54, 55, 56, 57, 60, 61, 62, 63, 64, 65, 68, 69, 72, 73, 74, 78, 79, 82, 83, 84, 86, 87, 88, 90, 91, 92, 94, 99, 100, 101, 102, 103, 107, 115
Describes limits used	100 (99.0)	18, 19, 20, 21, 22, 23, 24, 25, 26, 27, 28, 29, 30, 31, 32, 33, 34, 35, 36, 37, 38, 39, 40, 41, 42, 43, 44, 45, 46, 47, 48, 49, 50, 51, 52, 53, 54, 55, 56, 57, 58, 59, 60, 61, 62, 63, 64, 65, 66, 67, 68, 69, 70, 71, 72, 73, 74, 75, 76, 77, 118
States the process for selecting evidence included	100 (99.0)	18, 19, 20, 21, 22, 23, 24, 25, 26, 27, 28, 29, 30, 31, 32, 33, 34, 35, 36, 37, 38, 39, 40, 41, 42, 43, 44, 45, 46, 47, 48, 49, 50, 51, 52, 53, 54, 55, 56, 57, 58, 59, 60, 61, 62, 63, 64, 65, 66, 67, 68, 69, 70, 71, 72, 73, 74, 75, 76, 77, 118
Lists and defines all variables for which data was sought and any simplifications made	75 (74.3)	18, 19, 22, 23, 24, 27, 28, 29, 30, 31, 32, 33, 34, 35, 38, 40, 41, 42, 44, 45, 46, 48, 49, 51, 52, 53, 54, 55, 56, 60, 61, 62, 63, 65, 66, 67, 68, 69, 71, 72, 73, 74, 75, 77, 79, 80, 82, 84, 85, 86, 87, 89, 90, 91, 92, 94, 95, 96, 97, 100, 101, 102, 103, 104, 105, 106, 107, 109, 112, 113, 114, 115, 116
If done, provides a rationale for conducting critical appraisal of included sources	13 (12.8)	21, 73, 82, 100, 101
Described the methods of handing and summarising the charted data	86 (85.6)	18, 19, 20, 22, 23, 24, 25, 26, 27, 28, 29, 30, 31, 32, 33, 34, 35, 36, 37, 38, 39, 40, 41, 42, 44, 46, 48, 49, 50, 51, 52, 53, 54, 55, 56, 58, 60, 61, 62, 63, 64, 65, 66, 67, 68, 69, 70, 71, 73, 74, 75, 76, 77, 78, 79, 80, 82, 83, 84, 85, 86, 87, 88, 89, 90, 91, 92, 95, 96, 97, 99, 100, 101, 102, 103, 105, 106, 108, 109, 110, 112, 113, 115, 116, 117, 118
Provides the number of sources of evidence screened, assessed for eligibility, and included in the review ideally presented as a flow diagram	97 (96.0)	18, 19, 20, 21, 22, 23, 24, 25, 26, 27, 28, 29, 30, 31, 32, 33, 34, 35, 36, 37, 38, 39, 40, 41, 42, 43, 44, 45, 46, 47, 48, 49, 50, 51, 52, 53, 54, 55, 56, 57, 58, 59, 60, 61, 62, 63, 64, 65, 66, 67, 68, 69, 71, 72, 73, 74, 75, 76, 77, 78, 79, 80, 81, 82, 83, 84, 85, 86, 87, 88, 89, 90, 91, 92, 93, 94, 95, 96, 97, 98, 99, 100, 101, 102, 103, 104, 105, 106, 107, 109, 110, 112, 113, 114, 115, 116, 117, 118
For each evidence source, presents characteristics for which data were charted and provide citations	58 (57.4)	20, 21, 23, 27, 29, 38, 39, 40, 41, 45, 47, 48, 49, 50, 51, 52, 53, 55, 56, 58, 60, 61, 63, 65, 67, 68, 69, 71, 72, 73, 74, 75, 77, 79, 82, 84, 87, 89, 91, 92, 93, 96, 99, 100, 102, 106, 107, 114, 115, 116, 117, 118
If done, presents results of critical appraisal	13 (12.9)	21, 23, 34, 35, 36, 39, 59, 64, 65, 73, 82, 100, 101
For each included evidence source, present relevant data that were charted that related to the review questions and objectives	70 (69.3)	20, 21, 23, 27, 29, 31, 41, 44, 45, 46, 47, 48, 49, 50, 51, 52, 53, 55, 56, 58, 60, 61, 62, 63, 64, 65, 67, 68, 69, 71, 72, 73, 74, 75, 76, 77, 78, 79, 80, 82, 84, 85, 87, 89, 93, 95, 96, 99, 100, 102, 105, 106, 107, 113, 115, 116, 117, 118
Summarises charting results as they relate to research questions and objectives	97 (96.0)	18, 19, 20, 21, 22, 23, 24, 25, 26, 27, 28, 29, 30, 31, 32, 33, 34, 35, 36, 37, 38, 39, 40, 41, 42, 43, 44, 45, 46, 47, 48, 49, 50, 51, 52, 53, 54, 55, 56, 57, 58, 59, 60, 61, 62, 63, 64, 65, 66, 67, 68, 69, 70, 71, 72, 73, 74, 75, 76, 77, 78, 79, 80, 81, 82, 83, 84, 85, 86, 87, 88, 89, 90, 91, 112, 113, 114, 115, 116, 117, 118
Summarises the main results, linking to review questions and objectives	101 (100)	18, 19, 20, 21, 22, 23, 24, 25, 26, 27, 28, 29, 30, 31, 32, 33, 34, 35, 36, 37, 38, 39, 40, 41, 42, 43, 44, 45, 46, 47, 48, 49, 50, 51, 52, 53, 54, 55, 56, 57, 58, 59, 60, 61, 62, 63, 64, 65, 66, 67, 68, 69, 70, 71, 72, 73, 74, 75, 76, 77, 78, 79, 80, 81, 82, 83, 84, 85, 86, 87, 88, 89, 90, 91, 92, 93, 94, 95, 96, 97, 98, 99, 100, 101, 102, 103, 104, 105, 106, 107, 108, 109, 110, 111, 112, 113, 114, 115, 116, 117, 118
Discusses the limitations of the scoping review process	23 (22.8)	41, 42, 74, 75, 76, 84, 89, 90, 97, 101, 102, 103, 104, 107, 110, 114
Provides a general interpretation of the results with respect to the research questions/objectives	101 (100)	18, 19, 20, 21, 22, 23, 24, 25, 26, 27, 28, 29, 30, 31, 32, 33, 34, 35, 36, 37, 38, 39, 40, 41, 42, 43, 44, 45, 46, 47, 48, 49, 50, 51, 52, 53, 54, 55, 56, 57, 58, 59, 60, 61, 62, 63, 64, 65, 66, 67, 68, 69, 70, 71, 72, 73, 74, 75, 76, 77, 78, 79, 80, 81, 82, 83, 84, 85, 86, 87, 88, 89, 90, 91, 92, 93, 94, 95, 96, 97, 98, 99, 100, 101, 102, 103, 104, 105, 106, 107, 108, 109, 110, 111, 112, 113, 114, 115, 116, 117, 118
Describes funding sources of funding for the included sources of evidence	0 (0)	
Describes the role of the funders of the scoping review	38 (37.6)	19, 26, 28, 51, 53, 60, 62, 65, 66, 70, 72, 73, 74, 75, 80, 87, 88, 90, 92, 96, 97, 100, 101, 102, 105, 106, 109, 110, 112, 114, 115, 117

While the PRISMA‐ScR is quite detailed, we charted additional study details based on Arksey and O'Malley's framework as modified by Levac (See Table 2).^2^, ^5^ Most reviews described following Arksey and O'Malley's framework (n = 73, 72.3%)^2^, ^18^, ^19^, ^20^, ^21^, ^22^, ^23^, ^24^, ^25^, ^26^, ^27^, ^28^, ^29^, ^30^, ^31^, ^32^, ^33^, ^34^, ^35^, ^37^, ^39^, ^40^, ^41^, ^42^, ^44^, ^45^, ^46^, ^48^, ^49^, ^50^, ^53^, ^54^, ^55^, ^56^, ^58^, ^59^, ^62^, ^63^, ^64^, ^65^, ^66^, ^69^, ^70^, ^72^, ^74^, ^75^, ^77^, ^78^, ^79^, ^80^, ^83^, ^84^, ^86^, ^87^, ^88^, ^89^, ^90^, ^93^, ^95^, ^96^, ^97^, ^98^, ^99^, ^100^, ^101^, ^102^, ^103^, ^104^, ^105^, ^106^, ^111^, ^113^, ^114^, ^117^, ^118^ and 32 (31.7%)^22^, ^23^, ^26^, ^42^, ^44^, ^45^, ^46^, ^53^, ^54^, ^56^, ^62^, ^64^, ^69^, ^77^, ^78^, ^79^, ^83^, ^87^, ^90^, ^97^, ^100^, ^101^, ^104^, ^105^, ^113^, ^117^ of these reviews used Levac's revision.^5^

**TABLE 2:** Summary of scoping review characteristics charted based on Arksey and O'Malley's framework as modified by Levac

	Number studies (%)	References
Identifying relevant studies		
Conducted database searches using multiple databases	101 (100)	18, 19, 20, 21, 22, 23, 24, 25, 26, 27, 28, 29, 30, 31, 32, 33, 34, 35, 36, 37, 38, 39, 40, 41, 42, 43, 44, 45, 46, 47, 48, 49, 50, 51, 52, 53, 54, 55, 56, 57, 58, 59, 60, 61, 62, 63, 64, 65, 66, 67, 68, 69, 70, 71, 72, 73, 74, 75, 76, 77, 78, 79, 80, 81, 82, 83, 84, 85, 86, 87, 88, 89, 90, 91, 92, 93, 94, 95, 96, 97, 98, 99, 100, 101, 102, 103, 104, 105, 106, 107, 108, 109, 110, 111, 112, 113, 114, 115, 116, 117, 118
Hand‐searched included articles	72 (71.3)	20, 23, 24, 25, 27, 28, 29, 30, 36, 37, 39, 40, 41, 42, 43, 44, 46, 47, 48, 49, 50, 51, 52, 53, 54, 55, 56, 57, 58, 60, 61, 62, 63, 64, 65, 68, 70, 71, 72, 73, 74, 75, 76, 78, 79, 80, 81, 83, 84, 86, 88, 89, 90, 91, 92, 93, 96, 99, 100, 102, 104, 106, 107, 108, 110, 111, 113, 114, 116, 117, 118
Included a librarian	62 (61.3)	18, 19, 20, 21, 22, 23, 24, 29, 31, 32, 33, 34, 37, 38, 40, 41, 43, 44, 45, 46, 47, 48, 49, 51, 53, 54, 59, 60, 61, 62, 63, 66, 68, 69, 72, 73, 74, 75, 76, 77, 79, 80, 84, 88, 89, 90, 91, 92, 97, 100, 103, 104, 110, 112, 113, 115, 117, 118
Consulted article authors	4 (3.9)	28, 55, 71, 96
Selecting studies to be included		
Authors included physicians	55 (54.5)	18, 23, 24, 25, 26, 35, 40, 43, 44, 47, 48, 50, 52, 54, 55, 57, 59, 60, 61, 64, 67, 68, 73, 77, 80, 81, 82, 83, 85, 86, 87, 90, 94, 96, 98, 99, 100, 101, 102, 103, 105, 108, 109, 110, 112, 113, 116, 117, 118
Undergraduate Medical Education	37 (36.6)^a^	23, 25, 28, 50, 52, 54, 60, 61, 64, 68, 81, 83, 85, 86, 87, 90, 96, 98, 99, 100, 101, 102, 105, 107, 108, 109, 110, 112, 116, 117
Graduate Medical Education	31 (30.7)^a^	18, 23, 24, 40, 43, 44, 47, 48, 50, 55, 59, 64, 68, 73, 81, 82, 83, 94, 100, 103, 105, 108, 110, 113, 116
Continuing Medical Education	21 (20.8)^a^	18, 24, 28, 31, 32, 33, 48, 50, 57, 67, 68, 77, 80, 83, 100, 103, 105, 108, 110, 113, 118
Across all three levels	13 (12.9)	18, 100, 105, 108, 110
Authors included multiple health professions	44 (43.6)	19, 20, 21, 22, 41, 42, 45, 46, 49, 51, 53, 58, 62, 63, 65, 70, 71, 72, 74, 75, 78, 88, 91, 92, 95, 97, 104, 106, 111, 112, 114
Authors included physiotherapists only	2 (1.9)	69, 84
Authors included emergency medical technicians only	2 (1.9)	89, 93
Authors included nurses only	1 (1.0)	56
Data Charting		
Authors published data charting tool	36 (35.6)	19, 20, 24, 44, 45, 48, 53, 54, 55, 60, 61, 64, 68, 74, 79, 80, 83, 86, 87, 91, 95, 96, 100, 101, 103, 106, 107, 109, 112
Authors piloted their data charting tool	35 (34.6)	19, 22, 23, 24, 37, 44, 48, 50, 51, 53, 54, 55, 60, 61, 62, 63, 64, 67, 72, 73, 79, 80, 90, 92, 97, 99, 104, 105, 108, 113
Data charting was done by more than one author	65 (64.3)	18, 19, 21, 22, 23, 24, 25, 26, 29, 31, 32, 33, 34, 35, 36, 37, 38, 39, 40, 44, 45, 46, 48, 50, 51, 53, 55, 56, 60, 61, 62, 64, 65, 66, 67, 71, 72, 73, 74, 76, 77, 79, 80, 82, 83, 85, 86, 87, 90, 91, 92, 94, 95, 100, 103, 104, 105, 106, 107, 113, 115, 116, 117, 118
Collating and reporting results		
Critical appraisal conducted	13 (12.9)	21, 23, 34, 35, 36, 39, 59, 64, 65, 73, 82, 100, 101
Using Medical Education Research Study Quality Instrument	10 (9.9)	21, 23, 34, 35, 36, 59, 64, 73, 82, 100
Qualitative analysis conducted	61 (60.3)	18, 19, 20, 21, 22, 23, 24, 25, 31, 32, 33, 34, 39, 40, 44, 46, 49, 50, 56, 58, 60, 62, 63, 64, 65, 66, 67, 71, 74, 75, 76, 78, 79, 80, 83, 85, 86, 87, 90, 92, 95, 99, 100, 105, 106, 108, 109, 110, 111, 112, 113, 117
Using thematic analysis	38 (37.6)	20, 22, 23, 24, 25, 37, 39, 40, 44, 49, 58, 60, 62, 63, 64, 65, 67, 71, 74, 76, 78, 79, 83, 86, 90, 95, 99, 105, 108, 109, 110, 111, 112, 113
Using content analysis	10 (9.9)	21, 46, 66, 67, 78, 80, 85, 87, 110, 117
Using narrative analysis	5 (4.9)	29, 32, 50, 56, 92
Authors reported overall limitations	94 (93.0)	18, 19, 20, 21, 22, 23, 24, 26, 27, 28, 29, 30, 31, 32, 33, 34, 35, 36, 37, 38, 39, 40, 41, 42, 43, 44, 45, 46, 47, 48, 49, 50, 51, 52, 53, 54, 55, 56, 58, 59, 60, 61, 62, 63, 64, 65, 66, 67, 68, 69, 71, 72, 73, 74, 75, 76, 77, 78, 79, 80, 82, 83, 84, 85, 86, 87, 88, 89, 90, 91, 92, 93, 94, 95, 96, 97, 98, 99, 100, 101, 102, 103, 104, 105, 106, 107, 108, 110, 112, 113, 114, 115, 116, 118
Authors reported limitations of scoping review methodology	23 (22.8)	41, 42, 74, 75, 76, 84, 89, 90, 97, 101, 102, 103, 104, 107, 110, 114
Inability to conduct critical appraisal	14 (13.9)	41, 90, 101, 103, 107, 114
Consultations with key stakeholders		
Authors reached out to key stakeholders	20 (19.8)	21, 23, 28, 32, 39, 44, 45, 53, 55, 61, 64, 68, 70, 71, 73, 89, 93, 100, 106, 113

^a^ Several studies included combinations of learner levels thus counts sum to greater than 55 studies.

## 4 DISCUSSION

Scoping reviews are increasingly conducted in medical education and published by almost all of the core journals. Because scoping reviews aim to map the depth and breadth of emerging topics in the field and help clarify key concepts and definitions in the literature, we believe they have the *potential* to play a powerful role in the practice, policy and research of medical education. Additionally, because so many authors receive public funding for their reviews, and authors dedicate a substantial amount of internal resources (eg, faculty time, research assistant effort) to their conduct, it is critical that authors be good stewards of these resources by rigorously conducting and clearly reporting their work. With this in mind, and to move the field forward, we focus our discussion on areas we feel are ripe for improvement in the conduct and reporting of scoping reviews.

Researchers have highlighted the importance of authors linking their rationale to their research questions. Doing so helps to guide the scoping review's overall conduct, especially to inform the inclusion/exclusion of evidence and data extraction processes.^5^, ^13^ We observed some alignment in authors' rationales and their research questions/aims, but also noted room for improvement. For example, most authors' rationales and research questions mapped to those described by Arksey and O'Malley.^2^ However, Levac criticised these rationales as being applicable broadly to a variety of knowledge synthesis methodologies and not necessarily specific to scoping reviews.^5^ Thus, it is possible that this lack of specificity contributed to the suboptimal alignment between the reported rationales and research questions. As such, we encourage medical education researchers to consider whether or not Arksey and O'Malley's rationales are really ‘fit for purpose’ for the field of medical education. Additionally, editors and reviewers might want to ask scoping review authors to specifically describe *why* they selected a scoping review methodology and what factors influenced their decision to undertake a scoping review (eg, the nature of the literature, the intricacies of the topic, the expertise of their research team, and/or their personal needs such as a graduate student familiarising herself with a topic). More clearly articulating medical education‐focused rationales could help scholars (and journal reviewers) determine if a scoping review is right knowledge synthesis methodology and help make explicit the unique contribution of this review type for the field.

Author teams featured researchers from diverse backgrounds and multiple professions. Most teams included individuals with varied methodological training. This diversity can fundamentally impact the conduct and reporting of a scoping review.^13^ The same can be said for relatively homogeneous scoping review teams, whose implicit assumptions and epistemological positions or interpretations may also shape the review. Thus, with less than a quarter of author teams describing and reflecting on their team's characteristics, readers have limited opportunity to consider why and how certain decisions made in the review process may have influenced the review's conduct, findings and reporting. Taking a page from the qualitative research playbook, we encourage authors to include a brief reflexivity section in which they report and reflect on the characteristics of their team in relation to the study's design, data collection and analysis, and reporting.^120^ This information increases transparency and allows readers to make informed judgements about the conduct of the review, as well as its findings, interpretations and contributions to the field.

The inclusion of external stakeholders in research, including in knowledge syntheses, has been identified as a beneficial component of high‐quality, high‐impact research.^121^, ^122^ However, only a minority of included reviews described including external stakeholders. This finding suggests a missed opportunity to improve the execution and usefulness of medical education scoping reviews. To be fair, guidance on which stakeholders to include and how to include them in scoping reviews has been somewhat unclear.^122^ For example, Arksey and O'Malley have only suggested stakeholder inclusion,^2^ whereas Levac has declared it as essential.^5^ On the other hand, stakeholder consultation is absent from the PRISMA‐ScR.^8^ Despite this lack of clarity, several scoping review authors appear to be leveraging stakeholders in creative and critical ways. For example, one scoping review, which addressed education to reduce health gaps between Aboriginal and non‐Aboriginal peoples, integrated Aboriginal stakeholders throughout the entire conduct of the review.^106^ We suspect that this review would have suffered without stakeholder involvement. As we are unaware of any firm guidance on stakeholder inclusion in scoping reviews within medical education, we propose an important step forward would be for the field come to some consensus on best practice guidelines regarding the role of stakeholders. At the very least, review teams should make explicit why the stakeholders were involved and describe the ways in which the review was strengthened as a result of their input. Doing so could help to ensure scoping reviews are optimised for medical education.

Nearly half of the included authors chose to conduct a scoping review because of the nature of their topic or the available literature. Specifically, many of these authors commented on the heterogeneity of the literature and its emerging nature such that particular study designs (eg, randomised controlled trials) were unavailable for review. The ability to include multiple publication types and various materials is often seen as a hallmark of a scoping review. In fact, Arksey and O'Malley^2^ declared that ‘the whole point of scoping the field is to be as comprehensive as possible’. However, despite their stated rationales, multiple authors limited their inclusion criteria to empirical research and explicitly excluded heterogeneous works such as perspective articles, opinion pieces and innovations. In so doing, authors may have inadvertently (or advertently) missed work that is important for understanding an emerging research space. Moreover, six authors highlighted the heterogeneity of the included literature as a limitation. We can only guess as to why some authors made the choice to exclude some heterogeneous works (eg, lack of time, misunderstanding the point of a scoping review, etc); nonetheless, it does appear there may be some confusion regarding inclusion of various forms of knowledge and/or evidence (a point that has been discussed by Thomas et al).^13^ To this end, we propose that inclusion and exclusion criteria, including whether or not to include a variety of publication types and materials, should be driven by the research question(s). For example, in this scoping review, we were guided by research questions aimed at understanding the nature of scoping reviews in medical education. Thus, we did not include any other publication types because that approach would not allow us to answer our questions of interest.

Twelve reviews described critical appraisal of the articles they included. This contrasts with our finding that in 14 reviews (13.9%),^41^, ^90^, ^101^, ^103^, ^107^, ^114^ authors cited an inability to conduct an appraisal due to the nature of the scoping review methodology, which they described as a limitation. For example, one author wrote: ‘The nature of a scoping review eliminates any analysis of the quality of the research conducted, so the information supplied concerning the participants' comments regarding the usefulness of a peer‐coaching approach needs to be interpreted with caution’.^114^ In some cases, authors pointed to the heterogeneity of the literature as a barrier to critical appraisal, but in others there was a sense that in a scoping review critical appraisal is unpermitted. Similar to the inclusion of stakeholders, this appears to be a grey area with limited guidance. To our knowledge, there is no specific ‘rule’ that appraisal must or cannot be conducted in a scoping review. That said, the PRISMA‐ScR notes that authors should describe critical appraisal of included evidence if critical appraisal is done.^8^ Thus, we encourage researchers to consider their specific review in relation to their research questions and the nature of the literature included, and to then make an informed decision about incorporating (or not) critical appraisal.

As noted in Table 2, only two scoping reviews were registered and provided links to a submitted protocol.^35^, ^36^ Protocol registration increases transparency in review practices and has been associated with increased review quality.^123^ Additionally, protocol registration can help researchers avoid embarking on a review that is already underway. Currently, we are unaware of any medical education journal that requires or encourages protocol registration. This begs the question: is it finally time for the medical education community to have a serious conversation about the pros and cons of protocol registration?

### 4.1 Limitations of this scoping review

This study has several limitations. First, scoping reviews on other health professions education topics may have been missed because we focused only on a core set of medical education journals. For example, it is possible we missed scoping reviews focused on medical education topics, but that were published in clinical journals. Future research might consider expanding the sample of journals to more broadly survey the field. Additionally, because we directed our inclusion/exclusion criteria on authors' use of the term ‘scoping’ in the title or abstract, it is possible that we inadvertently excluded relevant scoping reviews in cases where the authors did not use that specific terminology. This limitation may have implications for earlier scoping reviews in which authors may have been less familiar with the term. Third, to guide our data extraction, we used the PRISMA‐ScR reporting guidelines, which was published in 2018. It is possible that authors publishing prior to 2018 were unaware of the importance of reporting many of the items in this reporting guideline, and thus did not include them (even if those data had been collected). However, PRISMA,^119^ which is the basis for the PRISMA‐ScR, was published in 2009 and contains many of the same items. This suggests that while the PRISMA is not specific to scoping reviews, authors should have had familiarity with most of these items. Lastly, we did not register our scoping review protocol. Although we do not view this oversight as a study limitation, per se, protocol registration is considered a best practice.^124^

## 5 CONCLUSION

Scoping reviews are increasingly conducted in medical education and published by almost all of the core journals examined here. Scoping reviews aim to map the depth and breadth of emerging topics; as such, they have the potential to play a critical role in the practice, policy and research of medical education. Although scoping reviews are not designed to result inaction‐oriented recommendations, per se, the results from the present study suggest that improvements are needed for this role to be fully realised. These findings suggest room for improvement in the conduct and reporting of scoping reviews, including the alignment of research questions with rationales for undertaking a review, the publishing of protocols and the inclusion of external stakeholders in published works.

## CONFLICT OF INTEREST

The authors declare that they have no conflict of interest.

## AUTHOR CONTRIBUTIONS

Lauren Maggio, Kelsey Larsen, Aliki Thomas, Joseph Costello, Anthony Artino made substantial contributions to the conception or design of the work; the acquisition, analysis, or interpretation of data for the work; the drafting of the work; its critical revision for important intellectual content; provided final approval of the version to be published; and agreed to be accountable for all aspects of the work in ensuring that questions related to the accuracy or integrity of any part of the work are appropriately investigated and resolved.

## ETHICAL APPROVAL

This research does not contain any human subjects.

## DISCLAIMER

The views expressed in this article are those of the authors and do not necessarily reflect the official policy or position of the Uniformed Services University of the Health Sciences, the Department of Defense, or the US Government.

## Supporting information

Appendix S1

Appendix S2

Appendix S3

Appendix S4

Appendix S5

## Data Availability

The data charting for all studies and the data charting tool has been deposited at: https://doi.org/10.6084/m9.figshare.12699698.v2
