# Supplementary figures and images for: Scoping reviews in medical education: A scoping review
Source: Med Educ. 2020 Dec 30;55(6):689–700. doi: 10.1111/medu.14431 (PMC8247025; doi:10.1111/medu.14431)

Appendix S2: A diagram of the inclusion process


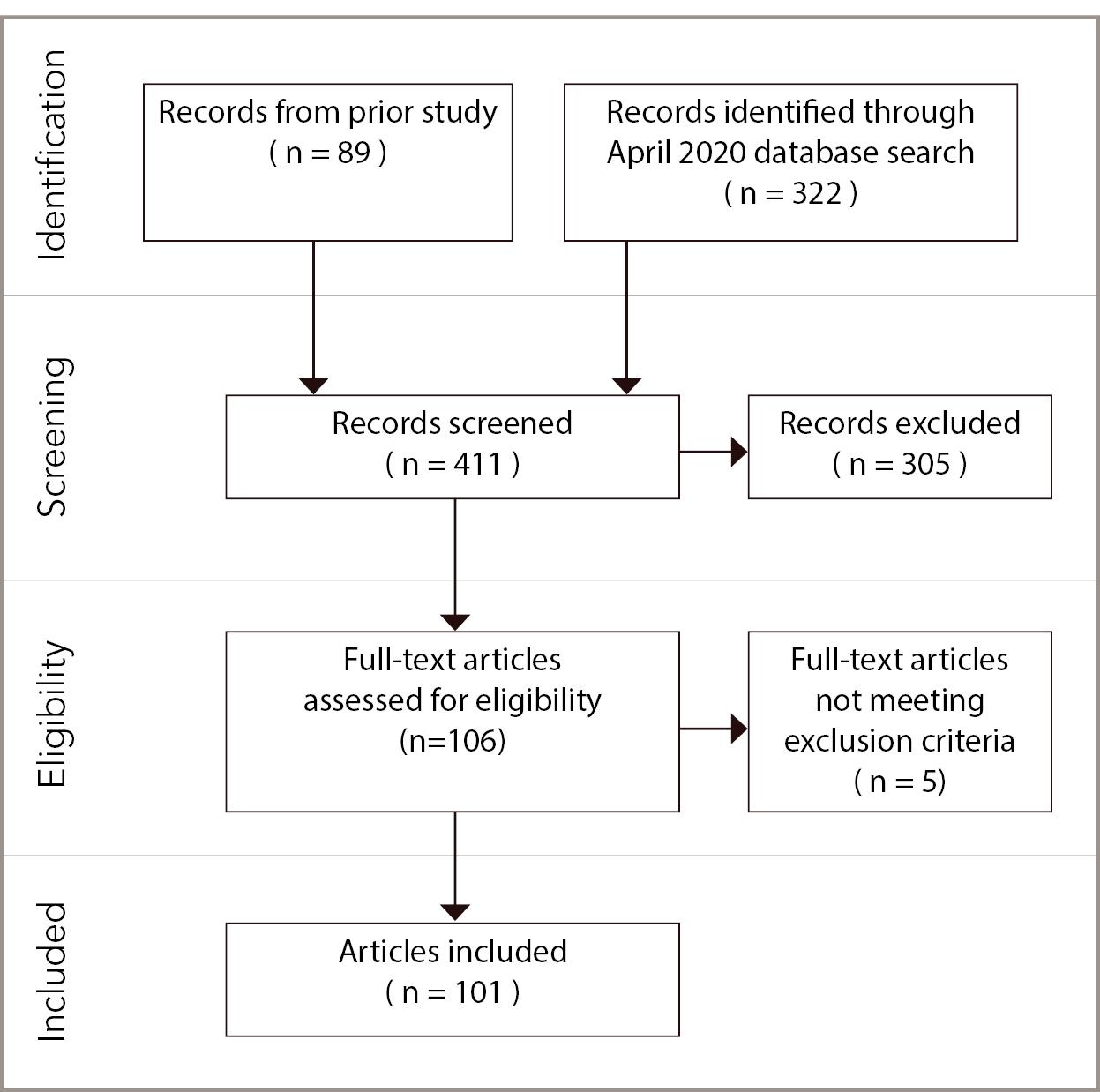

Supplement: Supplementary file 2 — Appendix S2 [file MEDU-55-689-s002.docx]
