# Supplementary material for: Scoping reviews in medical education: A scoping review
Source: Med Educ. 2020 Dec 30;55(6):689–700. doi: 10.1111/medu.14431 (PMC8247025; doi:10.1111/medu.14431)
Supplement: Supplementary file 3 — Appendix S3 [file MEDU-55-689-s004.docx]

Appendix S3:

Counts of Scoping Reviews in Medical Education published between 2011 and April 27, 2020


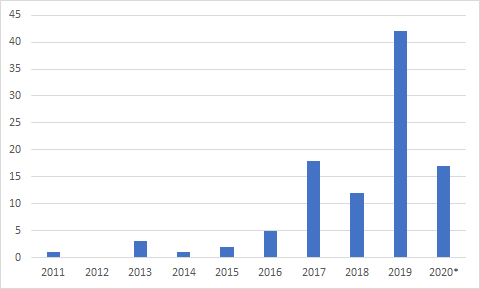


*2020 data includes only Scoping Reviews published January 1 - April 27, 2020 (i.e. 4 months).
