# Supplementary material for: Scoping reviews in medical education: A scoping review
Source: Med Educ. 2020 Dec 30;55(6):689–700. doi: 10.1111/medu.14431 (PMC8247025; doi:10.1111/medu.14431)
Supplement: Supplementary file 4 — Appendix S4 [file MEDU-55-689-s003.docx]

Appendix S4: Top 10 Cited Scoping Reviews published in core medical education journals between 2009-April 2020

| Title, Author, Publication Year | Number Citations |
| --- | --- |
| Humanism, the Hidden Curriculum, and Educational Reform: A Scoping Review and Thematic Analysis: Martimianakis, *Academic Medicine*, 2015. | 61 |
| Social Media Use by Health Care Professionals and Trainees: A Scoping Review: Hamm, *Academic Medicine,* 2013 | 59 |
| Feedback for Learners in Medical Education: What Is Known? A Scoping Review: Bing-You, *Academic Medicine,* 2017 | 37 |
| The Use of the Delphi and Other Consensus Group Methods in Medical Education Research: A Review: Humphrey-Murto, *Academic Medicine,* 2017 | 34 |
| Peer-coaching with health care professionals: What is the current status of the literature and what are the key components necessary in peer-coaching? A scoping review: Schwellnus, *Medical Teacher,* 2014 | 34 |
| Teaching Medical Error Disclosure to Physicians-in-Training: A Scoping Review: Stroud, *Academic Medicine,* 2013 | 32 |
| Mapping the dark matter of context: A conceptual scoping review: Bates, *Medical Education,* 2016 | 31 |
| Clarifying changes in student empathy throughout medical school: a scoping review: Ferreira-Valente, *Advances in Health Sciences Education: Theory and Practice*, 2017 | 24 |
| The Hidden Curricula of Medical Education: A Scoping Review: Lawrence, *Academic Medicine,* 2018 | 23 |
| Rethinking research in the medical humanities: a scoping review and narrative synthesis of quantitative outcome studies: Dennhardt, *Medical Education,* 2016 | 16 |
