# Supplementary material for: Scoping reviews in medical education: A scoping review
Source: Med Educ. 2020 Dec 30;55(6):689–700. doi: 10.1111/medu.14431 (PMC8247025; doi:10.1111/medu.14431)
Supplement: Supplementary file 5 — Appendix S5 [file MEDU-55-689-s001.docx]

Appendix S5: Rationales for conducting a scoping review and research questions in 101 scoping reviews published in 14 core medical education journals

| Rationales | For selecting scoping review methodology n=88  n (%) | References | Research question and/or study aims n=98 n (%) | References |
| --- | --- | --- | --- | --- |
| Summarize and disseminate research findings* | 77 (87.5) | 18-28,30-33,35-37,39-40,43-45,48-54,56,59-63,65-68,70,72,74-79,81,82,85-93,95,96,99-101,103-116,118 | 89 (90.1) | 18-32,34-39,41-69,71-74,76-82,84-88,92-94,96-103,105-107,109-114,116-118 |
| Examine the extent, range, and nature of research activity in a given area* | 74 (84.1) | 18-22,24-27,30-33,35-37,39,40,43,44,48-50,51-54,56,60,61,63,65-68,72,76-82,84-89,92,93,95,96,100,101,103-106,108-116,118 | 86 (87.8) | 18-30,32,34-39,41-57,59-69,71-74,76-82,84-88,92,94,96,97,99,101-107,109-118 |
| Contend with the nature of the study topic or available literature | 46 (52.3) | 19,21,23-27,31,35,37,39,40,41,45,50,54,58,59,61-64,68,73,75,77,80-82,86,92,94,99,101,104,105-107,109,111-114,116,118 | 18 (18.4) | 19,24,27,30,33,37,45,48,68,75,78,82,92,108,110,112,114,118 |
| Identify gaps in the existing body of literature* | 24 (27.3) | 18,20,26-28,30,33,59,60,61,63,70,80,84,91,95,96,98,101,104,111,112,115,118 | 13 (13.3) | 26,57,60,64,69,70,78,79,86,98,101,111,114 |
| Refine subsequent research inquiries (not including systematic reviews) | 15 (17.0) | 27,44,45,52,60,62,66,70,78,80,88,98,100,111,115 | 11 (11.2) | 22,32,36,50,52,61,67,70,72,,86,90 |
| Demonstrate the use of  theory or model implementation | 12 (13.6) | 51,53,56,58,60,67,68,95,98,103,104,116 | 7 (7.1) | 27,33,37,56,84,86,87 |
| Determine the value of undertaking a full     systematic review* | 5 (5.7) | 22,23,72,115,118 | 1 (1.1) | 72 |
| Assess quality or effectiveness | 5 (5.7) | 35,36,81,89,95 | 6 (5.9) | 25,35,36,81,89,95 |
| Create future products (e.g., a framework, curriculum, policy) | 4 (4.0) | 45,50,87,93 | 9 (8.9) | 25,45,47,68,74,82,88,97,98 |

*These rationales were described by Arksey and O’Malley (2005).
